# The impact of integrated disease management in high-risk COPD patients in primary care

**DOI:** 10.1038/s41533-019-0119-9

**Published:** 2019-03-28

**Authors:** Madonna Ferrone, Marcello G. Masciantonio, Natalie Malus, Larry Stitt, Tim O’Callahan, Zofe Roberts, Laura Johnson, Jim Samson, Lisa Durocher, Mark Ferrari, Margo Reilly, Kelly Griffiths, Christopher J. Licskai, The Primary Care Innovation Collaborative, Andrew Atkins, Bill Baker, Sara Dalo, Jean Piccinato, Denise Waddick, Brice Wong

**Affiliations:** 1 Asthma Research Group Windsor-Essex County Inc., Windsor, ON Canada; 2 Hotel-Dieu Grace Healthcare, Windsor, ON Canada; 3 Western University, London Health Sciences Centre, London, ON Canada; 4 Lawson Health Research Institute, London, ON Canada; 5 Amherstburg Family Health Team, Amherstburg, ON Canada; 6 Chatham Kent Family Health Team, Chatham, ON Canada; 7 Leamington Family Health Team, Leamington, ON Canada; 8 Windsor Family Health Team, Windsor, ON Canada; 9 Harrow Family Health Team, Harrow, ON Canada; 10 Tilbury Family Health Team, Tilbury, ON Canada; 11 Thamesview Family Health Team, Chatham, ON Canada

## Abstract

Patients with chronic obstructive pulmonary disease (COPD) have a reduced quality of life (QoL) and exacerbations that drive health service utilization (HSU). A majority of patients with COPD are managed in primary care. Our objective was to evaluate an integrated disease management, self-management, and structured follow-up intervention (IDM) for high-risk patients with COPD in primary care. This was a one-year multi-center randomized controlled trial. High-risk, exacerbation-prone COPD patients were randomized to IDM provided by a certified respiratory educator and physician, or usual physician care. IDM received case management, self-management education, and skills training. The primary outcome, COPD-related QoL, was measured using the COPD Assessment Test (CAT). Of 180 patients randomized from 8 sites, 81.1% completed the study. Patients were 53.6% women, mean age 68.2 years, post-bronchodilator FEV_1_ 52.8% predicted, and 77.4% were Global Initiative for Obstructive Lung Disease Stage D. QoL-CAT scores improved in IDM patients, 22.6 to 14.8, and worsened in usual care, 19.3 to 22.0, adjusted difference 9.3 (*p* < 0.001). Secondary outcomes including the Clinical COPD Questionnaire, Bristol Knowledge Questionnaire, and FEV1 demonstrated differential improvements in favor of IDM of 1.29 (*p* < 0.001), 29.6% (*p* < 0.001), and 100 mL, respectively (*p* = 0.016). Compared to usual care, significantly fewer IDM patients had a severe exacerbation, −48.9% (*p* < 0.001), required an urgent primary care visit for COPD, −30.2% (*p* < 0.001), or had an emergency department visit, −23.6% (*p* = 0.001). We conclude that IDM self-management and structured follow-up substantially improved QoL, knowledge, FEV1, reduced severe exacerbations, and HSU, in a high-risk primary care COPD population. Clinicaltrials.gov NCT02343055.

## Chronic lung disease: Integrated care shows promise

Patients enrolled on a chronic lung disease care program involving education and self-management report improved quality of life (QoL). People with severe forms of chronic obstructive pulmonary disease (COPD) often require urgent treatment for exacerbations and struggle with poor QoL. Christopher Licskai at Western University in Ontario, Canada, and co-workers trialed a one-year integrated disease management (IDM) program and compared it with standard care in 168 patients with severe COPD in primary care. The IDM involved regular collaboration with health professionals, guidance on best practice with medications, and personalized action plans. All participants completed the COPD Assessment Test, which indicated that IDM patients’ QoL scores improved during treatment, while QoL of those in standard care worsened over time. Significantly fewer IDM patients had severe exacerbations or the need for urgent medical care.

## Introduction

Chronic obstructive pulmonary disease (COPD) is a progressive lung disease characterized by increasing symptoms, decreasing (QoL), and increasing frequency of exacerbations.^1–4^ These inter-related patient outcomes are the foundational elements of the current Global Initiative for Chronic Obstructive Lung Disease (GOLD) severity classification (A–D).^1^ GOLD A and B patients are low and medium-risk patients who infrequently experience exacerbations.^1^ Collectively, GOLD C and D patients are high-risk patients defined by frequent exacerbations and/or a severe exacerbation requiring hospitalization.^1^ The high-risk “frequent-exacerbation” COPD phenotype persists over time,^5^ and accounts for one-third of the COPD patient population.^2^ Exacerbations exact a substantial personal toll on COPD patients, reducing their QoL significantly.^6–8^ In addition, COPD-related hospitalization accounts for more than half the cost of managing COPD in our health systems.^9,10^ International practice guidelines recommend effective pharmacologic and non-pharmacologic interventions to specifically address these patient and health system outcomes^1,11,12^; however, the impact of these recommendations on high-risk COPD patients in our communities has been limited by a substantial knowledge-to-care implementation gap.

The majority of COPD patients are managed by primary care practitioners.^13,14^ Although evidence-based management of COPD is increasingly complex, primary care providers manage high-risk COPD patients with multiple comorbidities within health systems that have enduring challenges. There are diagnostic barriers related to spirometry access and utilization.^15–18^ There is a low level of provider knowledge of COPD clinical practice guidelines.^15–18^ Chronic management of severe COPD requires multiple medications provided in different inhalation devices. To achieve self-efficacy, patients require self-management education and ongoing support. In practical terms, these evidence-based objectives are difficult to achieve by individual practitioners within the context of a regular clinical encounter. Thus, in practice, a minority of patients have an objectively confirmed diagnosis, or action plan, receive smoking cessation counseling, and for many medications, are under-prescribed relative to disease severity.^15–22^ Narrowing the knowledge-to-care implementation gap in primary care requires transformative innovation.

Evidence suggests that integrated disease management (IDM), which utilizes a team care model that supports physicians and patients to improve best-practice implementation, may be a transformative approach. The team care model can narrow the knowledge-to-care implementation gap and concurrently improve health outcomes in COPD.^23^ IDM has been defined as “a group of coherent interventions designed to prevent or manage one or more chronic conditions using a systematic, multidisciplinary approach, and potentially employing multiple treatment modalities”.^24^ The goal of chronic disease management is “to identify persons at risk… to promote self-management by patients, and to address the illness… with maximum clinical outcome, effectiveness, and efficiency.”^24^ IDM includes the “collaborative self-management” or “supported self-management” currently recommended by international guidelines.^1,6,11,12^ These strategies include a patient action plan to support early intervention to mitigate the impact of severe exacerbations on symptoms and QoL.

A recent meta-analysis on COPD IDM in a variety of healthcare settings concluded that IDM improved QoL, exercise capacity, and reduced hospitalization.^23^ However, IDM interventions are not uniform; they are complex interventions with a wide variety of components. Therefore, study authors performed a sub-set analysis including the following IDM sub-types: IDM-exercise predominant (pulmonary rehabilitation), IDM-self-management predominant, and IDM-structured follow-up. In the sub-set analysis, they were only able to confirm the efficacy of IDM-exercise predominant (pulmonary rehabilitation). Following publication of the metanalysis, researchers in the Netherlands published two more primary care randomized controlled trials (RCTs) examining IDM-self-management predominant interventions. Citing certain limitations, both studies failed to demonstrate a differential improvement in QoL.^25,26^ Thus, there is no clear evidence that IDM-self-management predominant or IDM-structured follow-up interventions are efficacious. There is, however, strong evidence of the efficacy of IDM in COPD in general, and specific efficacy of IDM-exercise predominant (pulmonary rehabilitation) interventions. We assert, therefore, that there remains an opportunity to develop and evaluate the efficacy of IDM-self-management and IDM-structured follow-up interventions in primary care.

In this study, our objective was to develop a COPD IDM-self-management and COPD IDM-structured follow-up intervention in primary care, including: patient identification, accurate diagnosis, case management, patient education, and skills training, and then to evaluate the IDM intervention in a high risk, frequent exacerbation population with a poor baseline QoL. We hypothesized that IDM would improve the QoL of high-risk, exacerbation-prone COPD patients, compared to a usual care control group.

## Results

### The IDM program intervention

Intervention subjects received on-site spirometry, case management, education, and skills training, including self-management education by a certified respiratory educator (CRE) at baseline (1 h), 3 months post-enrollment (45 min), and either a telephone contact or in-person visit at 6 and 9 months (15–30 min). All visits occurred in the primary care practice where the individual normally received care. The CREs involved were all regulated healthcare professionals whose scope of practice included patient counseling and who have successfully completed a Canadian Network for Respiratory Care approved respiratory educator program.^27^ The CREs that were COPD certified for this project were experienced asthma educators who provided services in an established primary care asthma program. During patient encounters, CREs were supported by a scalable electronic point-of-service system (POSS) developed for the project that guided them through the standardized evidence-based interventions and recorded all care elements delivered (Supplement 1). The IDM intervention identified patient-specific goals and emphasized shared decision making. The specific elements of IDM are categorized under case management, education, and skills training, and summarized in Table 1.

**Table 1:** Components of integrated disease management

	Number (proportion) receiving the intervention (*n* = 72)
*Case management*	
General patient support, education, and skills management training	72 (100%)
Prednisone and/or antibiotic prescription for exacerbation management	42 (58.3%)
Patient received Influenza vaccination during the study year	65 (90.3%)
Patient has received pneumococcal vaccination within the past 10 years or during the study year	63 (87.5%)
Referral to health professionals as needed	22 (31%)
Smoking cessation counseling provided or referral made to a cessation program	36 (50%)
*Educational topics*	
Understanding the meaning of a COPD diagnosis. Basic COPD pathophysiology	70 (97.2%)
Strategies for energy conservation	62 (86.1%)
Importance of regular exercise	69 (95.8%)
Nutrition counseling	36 (50%)
Role of and correct use of COPD medications and the importance of medication adherence	71 (98.6%)
Travel planning	19 (26.4%)
Advanced care/end-of-life planning	18 (25%)
*Skills training*	
Self-management education including flare-up/exacerbation awareness and management. When to initiate prednisone OR prednisone and antibiotic. (Including a written action plan)	72 (100%)
Patient used their action plan	69 (95.8%)
Patient was confident using their action plan	52 (72.2%)
Inhaler device technique	72 (100%)
Coping skills	47 (65.3%)
Breathing techniques	72 (100%)

The final management plan for each in-person visit was confirmed by the primary care physician during a 5–7 min encounter immediately following the CRE evaluation.

### Usual care

Patient care was delivered according to normal practice patterns in the Family Health Teams (FHTs). COPD care in Canada is usually delivered on an “as needed” or “needs to be assessed” basis. Study visits (no defined clinical intervention) in the usual care arm which were for the purpose of measuring study outcomes only, were scheduled at the same intervals as the IDM visits at baseline, 3, 6, and 9 months, with the close-out visit at one year.

### Study subjects

**Fig. 1:**
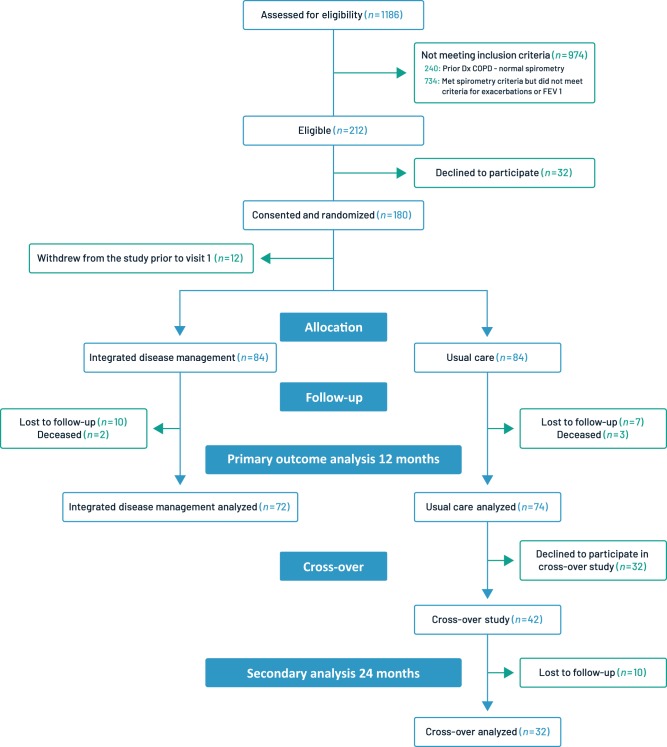
Patient flow diagram

Of 1186 subjects screened between November 2011 and January 2014, 974 were deemed ineligible (240 [20.2%] with normal spirometry [excluding COPD] and 734 [61.9%] who did not meet exacerbation criteria or had a predicted FEV ≥ 70%), leaving 212 eligible patients, of whom 32 declined to participate (Fig. 1). Of the 180 who were randomized, 12 withdrew prior to visit 1, leaving 168 study participants. A total of 22 subjects were lost to follow-up, 12 within the IDM arm and 10 within the usual care arm. Out of the 74 usual care subjects who completed the primary one-year study, 42 (56.8%) agreed to cross-over to receive IDM and 32 (76.2%) completed the 12 months cross-over analysis (24 months after initial enrollment).

Population demographics, baseline clinical and physiologic characteristics, and health service utilization (HSU) in the prior year are presented in Table 2 by treatment group. There were 53.6% women, mean age 68.2 years, forced expiratory volume in one second (FEV1) 52.8% predicted, 77.4% GOLD stage D, and baseline COPD Assessment Test (CAT) score 21.1 (standard deviation [SD] 7.2). Patient characteristics were generally well balanced between the treatment groups. In the IDM group, there were numerically more women and more GOLD D patients. The usual care arm had a numerically lower baseline CAT score (better QoL) and more smokers.

**Table 2:** Demographics and baseline clinical characteristics

	All subjects (*n* = 168)	Integrated disease management (*n* = 84)	Usual care (*n* = 84)
Sex, female	90 (53.6%)	50 (59.5%)	40 (47.6%)
Age, years	68.2 (9.7)	68.6 (9.6)	67.9 (9.8)
Caucasian	164 (97.6%)	82 (97.6%)	82 (97.6%)
BMI (kg/m^2^)	27.3 (6.3)	27.9 (6.9)	26.8 (5.6)
*FEV1*			
Pre-bronchodilator—litres	1.39 (0.54)	1.38 (0.55)	1.40 (0.53)
Pre-bronchodilator—% predicted	52.8 (14.5)	53.6 (14.2)	52.0 (14.7)
Post-bronchodilator—litres	1.43 (0.52)	1.42 (0.54)	1.43 (0.51)
Post-bronchodilator—% predicted	54.3 (14.6)	55.5 (14.5)	53.2 (14.7)
*FEV1/FVC ratio*			
Post-bronchodilator	54.6 (11.1)	55.6 (11.8)	53.6 (10.4)
Current smoker	81 (48.2%)	33 (39.3%)	48 (57.1%)
Baseline MRC score (range 0–5)	2.9 (0.9)	3.0 (0.9)	2.8 (0.8)
*GOLD stage*			
(a) A	4 (2.4%)	3 (3.6%)	1 (1.2%)
(b) B	30 (17.9%)	12 (14.3%)	18 (21.4%)
(c) C	4 (2.4%)	0 (0.0%)	4 (4.8%)
(d) D	130 (77.4%)	69 (82.1%)	61 (72.6%
Baseline CAT score (range 0–40)	21.1 (7.2)	22.8 (7.2)	19.5 (6.9)
Severe exacerbation (prior year: prednisone and/or antibiotics)	126 (75.0%)	63 (75.0%)	63 (75.0%)
*COPD health service use prior year*			
(a) Number of urgent physician visits	2.1 (1.9)	2.4 (2.1)	1.9 (1.5)
Any urgent physician visit	141 (83.9%)	72 (85.7%)	69 (82.1%)
(b) Number of ED visits	0.5 (0.9)	0.6 (0.8)	0.5 (1.0)
Any ED visit	59 (35.1%)	36 (42.9%)	23 (27.4%)
(c) Number of hospitalizations	0.3 (0.6)	0.3 (0.7)	0.3 (0.5)
Any hospitalization	34 (20.2%)	14 (16.7%)	20 (23.8%)

Data presented as frequency (%) or mean (standard deviation)

*BMI* body mass index, *CAT* COPD assessment test, *COPD* chronic obstructive pulmonary disease, *ED* Emergency Department, *FEV1* forced expiratory volume in one second, *FVC* forced vital capacity, *GOLD* Global Initiative for Chronic Obstructive Lung Disease, stage based on 2016–2017 criteria

### Process/intermediate outcomes—IDM

All study patients (IDM and control) had their clinical diagnosis of COPD confirmed by pre-bronchodilator and post-bronchodilator spirometry. In the IDM, CREs achieved standardized elements of case management, education, and skills training in a high proportion of patients (Table 1). All IDM patients received inhaler device instruction, all patients had a written self-management action plan, 95.8% reported using their plan, 58.3% had a prescription at home for prednisone and/or antibiotics to self-activate if needed, and 72.2% reported they were confident using their plan.

### Primary outcome: COPD specific QoL—COPD assessment test (CAT) score

QoL improved in the IDM cohort with a CAT score of 22.6 (SD 6.8) at baseline and 14.8 (SD 6.0) at 12 months. The CAT score declined in the usual care arm from 19.3 (SD 7.3) to 22.0 (SD 6.6). The adjusted difference between IDM and usual care at 12 months was 9.3 (95% confidence interval [CI] 7.8–10.8 [*p* < 0.001]) (Table 3). When, as a sensitivity analysis, a constrained longitudinal model was used to analyze CAT scores, the between-group difference was found to be significant (*p* < 0.001).

**Table 3:** Main outcomes: within and between group differences at 12 months

	Integrated disease management (*n* = 72)			Usual care (*n* = 74)			Between-group difference	
	Baseline	12 months	Change from baseline	Baseline	12 months	Change from baseline	Adjusted difference (95% CI)	*p*-Value
*COPD assessment test (CAT, 0–40)*								
Mean	22.6 (6.8)	14.8 (6.0)	−7.8 (5.1)	19.3 (7.3)	22.0 (6.6)	2.7 (5.0)	9.3 (7.8, 10.8)	<0.001
Proportion with CAT improvement >3	–	–	63/72 (87.5%)	–	–	4/74 (5.4%)	82.0% (72.7%, 91.4%)	<0.001
*Clinical COPD Questionnaire (CCQ, 0–6)*								
Symptoms	3.26 (1.28)	2.19 (1.12)	−1.07 (1.03)	2.82 (1.39)	3.16 (1.36)	0.34 (1.02)	1.27 (0.97, 1.57)	<0.001
Function	2.87 (1.34)	1.92 (1.29)	−0.95 (1.22)	2.15 (1.27)	2.55 (1.30)	0.41 (0.97)	1.10 (0.77, 1.44)	<0.001
Mental	2.92 (1.83)	1.24 (1.45)	−1.67 (1.69)	2.32 (1.76)	2.53 (1.79)	0.21 (1.45)	1.61 (1.17, 2.04)	<0.001
Total	3.04 (1.20)	1.89 (1.07)	−1.14 (1.01)	2.45 (1.28)	2.79 (1.28)	0.34 (0.93)	1.29 (1.00, 1.58)	<0.001
*Bristol knowledge questionnaire*								
Total (%)	41.0 (16.2)	74.9 (18.5)	33.9 (14.2)	41.9 (13.8)	46.2 (16.6)	4.4 (15.4)	29.6 (24.9, 34.2)	<0.001
*FEV1*								
Pre-bronchodilator—litres	1.45 (0.57)	1.52 (0.66)	0.07 (0.24)	1.42 (0.50)	1.40 (0.53)	0.02 (0.21)	0.10 (0.02, 0.18)	0.016
Pre-bronchodilator—% predicted	54.5 (13.9)	57.1 (17.1)	2.6 (9.3)	52.7 (15.3)	51.9 (16.3)	−0.8 (7.5)	3.5 (0.4, 6.5)	0.025
*FEV1/FVC*								
Pre-bronchodilator	55.8 (13.8)	57.3 (12.3)	1.5 (7.6)	53.7 (10.3)	53.3 (11.2)	−0.4 (5.0)	2.2 (0.1, 4.5)	0.044
*Severe exacerbations (prednisone and/or antibiotics)*								
Proportion: One or more severe exacerbations	60 (83.3%)	24 (33.3%)		54 (73.0%)	61 (82.4%)		−48.9% (−62.5%, −35.3%)	<0.001
*Health services utilization*								
Number of urgent physician visits/year	2.43 (2.23)	0.71 (0.97)	−1.72 (2.35)	1.96 (1.58)	2.16 (2.16)	0.20 (2.68)	1.46 (0.90, 2.02)	<0.001
Proportion: one or more urgent physician visits	60 (83.3%)	30 (41.7%)		60 (81.1%)	53 (71.6%)		−30.2% (−45.7%, −14.7%)	<0.001
Number of ED visits/year	0.57 (0.84)	0.17 (0.44)	−0.40 (0.93)	0.47 (1.01)	0.47 (0.71)	0.00 (1.11)	0.31 (0.12, 0.51)	0.002
Proportion: one or more ED visits	30 (41.7%)	10 (13.9%)		20 (27.0%)	28 (37.8%)		−23.6% (−37.3%, −9.9%)	0.001
Number of hospitalizations year	0.26 (0.65)	0.15 (0.55)	−0.11 (0.83)	0.23 (0.45)	0.26 (0.64)	0.03 (0.62)	0.10 (−0.09, 0.29)	0.305
Proportion: one or more hospitalizations	13 (18.1%)	8 (11.1%)		16 (21.6%)	13 (17.6%)		−5.8% (−17.8%, 5.1%)	0.283

*COPD* chronic obstructive pulmonary disease, *ED* Emergency Department, *FEV1* forced expiratory volume in one second, *FVC* forced vital capacity

### COPD-specific QoL—Clinical COPD Questionnaire (CCQ) score

Total CCQ score and scores in all domains improved in the IDM group. There was no improvement in the usual care control group. The adjusted difference between IDM and usual care at 12 months for the total score was 1.29, mental domain 1.61, functional domain 1.1, and symptom domain 1.27 (all *p* < 0.001) (Table 3).

### COPD knowledge—Bristol knowledge questionnaire

The IDM cohort improved their COPD knowledge more than the usual care control group in the total score by 29.6% (*p* < 0.001) (Table 3), and in each of the topic domain scores (Table 4).

**Table 4:** Bristol knowledge questionnaire

	Integrated disease management (*n* = 72)			Usual care (*n* = 74)			Between-group difference	
	Baseline	12 Months	Change from baseline	Baseline	12 Months	Change from baseline	Adjusted difference (95% CI)	*p*-Value
*Knowledge domain*								
Epidemiology	34.3 (20.8)	72.3 (26.0)	38.0 (26.0)	37.8 (19.6)	38.9 (21.6)	1.1 (24.9)	35.2 (28.0, 42.5)	<0.001
Etiology	54.3 (28.9)	86.0 (17.8)	31.7 (23.5)	58.9 (30.5)	60.0 (25.8)	1.1 (29.0)	28.0 (21.5, 34.4)	<0.001
Symptoms	54.3 (26.8)	85.7 (23.1)	31.4 (30.6)	55.1 (23.3)	63.0 (27.0)	7.9 (27.0)	23.7 (15.9, 31.5)	<0.001
Breathlessness	38.9 (24.3)	79.1 (26.5)	40.3 (31.5)	38.6 (19.1)	45.3 (24.9)	6.7 (25.1)	34.2 (26.0, 42.4)	<0.001
Phlegm	45.4 (26.4)	79.1 (22.2)	33.7 (25.1)	52.3 (22.5)	50.7 (29.3)	−1.6 (28.4)	32.0 (24.3, 39.7)	<0.001
Infections	37.1 (27.9)	73.7 (19.7)	36.6 (26.8)	44.1 (24.5)	46.6 (26.0)	2.5 (25.8)	29.9 (22.8, 37.0)	<0.001
Exercise	57.1 (27.5)	87.7 (21.1)	30.6 (29.0)	51.0 (23.3)	61.1 (29.0)	10.1 (33.4)	25.6 (17.3, 33.9)	<0.001
Smoking	58.0 (22.1)	73.1 (19.5)	15.1 (22.7)	58.6 (22.9)	63.3 (23.8)	4.7 (23.5)	10.5 (4.0, 17.0)	0.002
Vaccination	50.6 (25.4)	80.9 (20.3)	30.3 (21.5)	55.3 (25.1)	60.0 (23.1)	4.7 (24.8)	22.9 (16.9, 29.0)	<0.001
Inhaled bronchodilators	30.3 (28.2)	66.6 (31.3)	36.3 (28.1)	26.6 (23.6)	33.7 (25.4)	7.1 (22.5)	31.2 (23.6, 38.8)	<0.001
Antibiotics	33.1 (25.0)	66.9 (24.8)	33.7 (24.9)	34.5 (25.9)	37.3 (28.0)	2.7 (26.5)	30.6 (23.0, 38.1)	<0.001
Oral steroids	24.9 (26.9)	68.0 (34.5)	43.1 (31.7)	20.0 (26.2)	22.7 (26.5)	2.7 (30.2)	43.3 (34.4, 52.3)	<0.001
Inhaled steroids	14.6 (21.0)	54.3 (33.0)	39.7 (32.6)	11.8 (19.4)	18.9 (26.0)	7.1 (24.8)	34.5 (25.4, 43.6)	<0.001
Total	41.0 (16.2)	74.9 (18.5)	33.9 (14.2)	41.9 (13.8)	46.2 (16.6)	4.4 (15.4)	29.6 (24.9, 34.2)	<0.001

### Lung function

There was no significant change from baseline to 12 months in the pre-bronchodilator FEV1 in the usual care cohort. At 12 months, the IDM cohort had a mean increase in pre-bronchodilator FEV1 of 100 ml (*p* = 0.016) (Table 3).

### Severe exacerbation and health service utilization (HSU)

The proportion of IDM patients experiencing a severe COPD exacerbation was 48.9% lower than usual care patients (*p* < 0.001). Similarly, 30.2% fewer IDM patients required an urgent physician visit and 23.6% fewer patients required an emergency department (ED) visit for COPD exacerbation (*p* < 0.001 and *p* = 0.001, respectively). The proportion of subjects hospitalized was numerically lower in the IDM group; however, this between-group difference was not statistically significant (Table 3).

### Cross-over from usual care to IDM

Patients who crossed-over from usual care to IDM improved their CAT score from a baseline of 22.2 (SD 6.7) to 14.2 (SD 8.3) 12 months following cross-over, a difference of 8.0 (SD 5.4) (*p* < 0.001). The CCQ total score and the domain scores all improved, decreasing from total score 2.58 (SD 1.15) to 1.65 (SD 0.99), symptom domain 2.97 (SD 1.23) to 2.11 (SD 1.20), function domain 2.31 (SD 1.17) to 1.53 (SD 1.04), and mental domain 2.34 (SD 1.87) to 0.94 (SD 1.20) (all *p* < 0.001).

## Discussion

Internationally, this is the first study of a COPD IDM-self-management and COPD IDM-structured follow-up intervention in primary care, to demonstrate that IDM substantially improves COPD-related QoL. IDM also improved a secondary QoL measure (CCQ), FEV1, and COPD-related knowledge. Additionally, we demonstrated that fewer IDM patients had severe exacerbations that required an urgent visit to their primary care physician or an ED visit. This Canadian study in primary care complements the seminal work of Bourbeau and colleagues, who demonstrated improvements in QoL in a recently hospitalized severe COPD population in a Canadian specialty care setting.^28^

The results of this study are consistent with those reported in a recent systematic review on IDM in COPD.^23^ Kruis and colleagues summarized the world literature analyzing 26 trials involving 2997 people from 11 countries in a variety of health care settings, concluding that IDM improved QoL, exercise capacity, and reduced hospitalization.^23^ Although the authors confirmed this finding in a primary care sub-analysis with five studies, only one study in the metanalysis evaluated an IDM-self-management predominant intervention, and that study was negative. The other four studies in the primary care subset metanalysis were IDM-exercise predominant (pulmonary rehabilitation) interventions that confirm the QoL benefit of pulmonary rehabilitation in primary care. Our study findings are complementary in that they confirm the benefit of IDM with a focus on self-management and structured follow-up in primary care.

A direct comparison of the magnitude of effect between our study and those in the Kruis systematic review is complicated by the use of different QoL tools. We used the CAT score to measure QoL in this study because it is a concise, reliable, and validated instrument that is easily implemented in primary care.^29,30^ The St. George’s Respiratory Questionnaire (SGRQ) and the CAT score perform similarly, but they operate on different numeric scales (0–100 vs. 0–40) and have different minimum clinically important differences (MCIDs).^29–32^ The QoL difference between IDM and usual care in our study was CAT 10.4 (MCID 3). In the Kruis metanalysis, the mean improvement in QoL measured by the SGRQ in the primary care sub-set was 4.68 (MCID 4).^23^ Conservatively, the improvement in QoL measured in our study is consistent with the improvements reported in primary care IDM-pulmonary rehabilitation predominant interventions. Although pulmonary rehabilitation is the first choice in patients with a poor baseline, IDM-self-management and IDM-structured follow-up may be an option for patients where pulmonary rehabilitation is not available, or for patients who are unable or unwilling to participate in an exercise program.

Since the publication of the systematic review, and following this study’s initiation, two primary care RCTs evaluating COPD IDM-self-management interventions in the Netherlands have reported negative results.^25,26^ Bischoff and colleagues allocated 165 subjects equally into three arms: self-management, including the Canadian “Living Well with COPD” program, written self-management action plan, education, and case management, or routine monitoring, or usual care.^25^ Neither of the intervention arms had a differential impact on QoL. In another study, Kruis and colleagues enrolled 1086 patients from 40 primary care practices into a cluster RCT comparing IDM to usual care based on international guidelines, and found no difference in QoL.^26^ Collectively the authors in these studies suggest that the failure to find a differential benefit in favor of IDM may be explained by the overall high quality of guideline-based care provided by general practitioners in the Netherlands, a COPD population with a relatively good baseline QoL (ceiling effect), incomplete implementation of the targeted interventions (mean < 50%),^33^ and heterogeneity in the skill set of the community practical nurses delivering the intervention.^25,26^ By contrast, our study was conducted in a health system where a knowledge-to-care gap implementation gap exists, we recruited a severe exacerbation-prone COPD population (GOLD C and D > 80%) with a poor baseline QoL (no ceiling effect), and our patients were supported by specialized experienced CREs. One specific comparison that supports the ceiling effect hypothesis as an explanation for the negative finding in the Kruis RCT is the difference in the baseline CCQ in the Kruis study compared to our study. The CCQ in the Kruis population was 1.5 at baseline, whereas the CCQ in our population was 3.04, indicating a comparatively lower baseline QoL.

The Dutch experience highlights the need for robust implementation and training strategies to facilitate the spread and scale of successful regional programs nationally. This study was conducted in Ontario, Canada’s most populous province with >13 million people, >850,000 people with COPD, and a universal health care system.^34^ In this study, we demonstrated effective regional implementation delivering IDM in eight sites across 4233 square kilometers. We supported implementation with an electronic POSS used by CREs as a component of the encounter (Supplement 1). The POSS was developed to standardize the intervention based on clinical practice guidelines, and to prompt action on pre-determined case management, education, and skills training objectives. These standard interventions were tracked as intermediate/process outcomes within the POSS, permitting an analysis that confirmed the interventions were delivered to nearly all patients (Table 1). Morganroth also reported performance improvement in primary care clinics following the implementation of a web-based COPD disease management system.^35^ Leveraging the POSS will strengthen future plans to spread and scale this effective IDM intervention across the health system.

Some limitations of this study should be noted. First, the nature of the intervention did not permit a double blind design; thus, we cannot exclude the possibility of performance bias. Study questionnaires were administered by personnel who were aware of the patient assignment. We mitigated potential detection bias by selecting simple validated questionnaires that were completed by the patients independent of the influence of study personnel, specifically by allocating time for independent completion at the beginning of the study visit, and by carefully instructing study personnel that the patient must complete the questionnaires independently. Improvement in several secondary outcomes that are not affected by detection bias, such as severe exacerbations, FEV1, and HSU suggest a limited impact of detection bias in the study. We analyzed our data using a complete case analysis approach. As a sensitivity analysis, we performed a constrained longitudinal data analysis on the CAT score confirming the primary outcome.

IDM is a complex intervention, and as such, it was not possible to identify the specific intervention(s) or the mechanism leading to improved QoL; however, reasonable inferences can be made. First, case management with regular clinical review and a self-management action plan were effectively delivered in this study, and collectively are known to improve COPD-related QoL.^36^ Secondly, we reported improvements in the mental status and activity domains of the CCQ, which are directly correlated to QoL.^9^ The improvement in the mental domain in our study may have been related to patients having access to a highly competent CRE case manager, to care that was planned rather than provided “as needed”, and to improved COPD-related knowledge. Thirdly, exacerbations significantly reduce QoL in COPD.^6–8^ Thus, preventing severe exacerbations in our population probably contributed to improved QoL. Upstream interventions that may have contributed to exacerbation prevention in our study include prescribing inhalers appropriate to disease severity, improved adherence, and/or better inhaler technique. Critical errors in inhaler device technique occur in 15.4–46.9% of activations.^37^ Although we did not directly track prescriptions, measure adherence, or objectively assess inhaler technique, IDM patients received case management, education, and skills training, which are likely to impact on adherence and inhaler device technique. It is reasonable to consider that the 100 mL improvement in FEV1 in the IDM group is a surrogate measure for these factors. Finally, improved exacerbation management may have also contributed to improved QoL. All patients received a written self-management action plan, the majority had a prescription for prednisone and/or antibiotics to self-activate, and nearly all patients reported using their plan.

We have not completed a health economic analysis, but report that the COPD program developed based on this study operates for $300 Canadian/patient/year, excluding physician costs. If the reduction in hospitalizations that was not statistically significant in this study is confirmed in a larger study population, we speculate that the acute care cost avoidance derived from this IDM intervention would make it very cost-effective. The economic model is expected to be most favorable in exacerbation-prone COPD populations with high acute HSU.

In a high-risk primary care population, we confirmed that an IDM-self-management and IDM-structured follow-up intervention substantially improved QoL, lung function, reduced severe exacerbations, and COPD-related urgent HSU. Key factors in the successful outcome of this study included objectively confirming a COPD diagnosis, identifying a high risk population, and engaging highly skilled CREs who provided standardized case management, education, and skills training interventions in the majority of patients. Future studies should include specific measures of patient self-efficacy, direct measurement of adherence, assess the concordance of inhaler prescription to therapeutic recommendations based on GOLD stage, formally assess inhaler device technique, and a health economic analysis. The spread and scale of successful regional programs to a national level will require an investment of resources to support additional team members, the careful monitoring of process outcomes and highly skilled staff operating within a robust continuous quality assurance program.

## Methods

### Study design

This was a multicenter study with a 12-month parallel group treatment design comparing COPD IDM patients to usual care patients. Patients with a diagnosis of COPD self-identified, were found by an electronic medical record (EMR) search of billing codes and physician-generated patient profiles, or during a scheduled visit. All patients underwent pre-bronchodilator and post-bronchodilator spirometry for diagnostic confirmation based on GOLD criteria.^1^ After obtaining informed consent, subjects were randomly assigned by opaque sealed envelopes in blocks of four, stratified by site, in a ratio of 1:1 to IDM or usual care. The randomization sequence was generated by our biostatistician. Study personnel were blinded to the sequence and blocking factor. ClinicalTrials.gov Identifier: NCT02343055.

### Study population

This study was conducted in a health system with a knowledge-to-care implementation gap, in four FHTs at eight sites, 50 physicians serving 100,000 patients, distributed regionally across 4233 square kilometers in Southwestern Ontario, Canada. FHTs are comprised of an interdisciplinary team of physicians, nurses, dieticians, and social workers. Most FHTs, including those participating in this study, do not normally have CRE team members.

We enrolled patients that were ≥40 years of age, current or ex-smokers with a minimum 10 pack year smoking history, had a post-bronchodilator FEV1 of ≤70% after four puffs of salbutamol and FEV1/forced vital capacity (FVC) ratio <0.7, and had a history of at least two exacerbations in the past 3 years or one exacerbation in the past year. An exacerbation was defined as a COPD worsening that required treatment with prednisone or antibiotics, or an urgent visit to a health care practitioner, an ED visit, or hospitalization. We excluded patients with a COPD exacerbation in the past 4 weeks, a diagnosis of asthma prior to the age of 40 years, use of long-term supplemental oxygen, a co-morbid illness that would interfere with study participation, scheduled for COPD rehabilitation, or a terminal illness. All patients provided written informed consent. Western University Health Science Research Ethics Board #18057E.

### Outcome measures

We measured key process outcomes as intermediate outcomes in the IDM arm to determine the frequency with which the planned case management, educational, and skills training occurred.

The primary outcome was the impact of IDM on COPD-related QoL. We measured QoL using the CAT score, ranging from 0 to 40, where higher scores reflect a lower QoL.^38^ The CAT score MCID has been reported to range between 1.2 and 4.0.^29–32,39^ We reported our CAT outcomes using an MCID of 3.

Secondary clinical and physiologic outcomes included QoL measured by the CCQ, ranging from 0 to 6, where lower scores indicate a better QoL;^40^ COPD-specific knowledge measured by the Bristol Knowledge Questionnaire, ranging from 0% to 100%, where higher scores reflect greater knowledge;^41^ airflow limitation measured by absolute and percent predicted FEV1 and FEV1/FVC ratio; the proportion of patients experiencing a COPD exacerbation defined as a sustained worsening requiring prednisone and/or antibiotics; and the proportion and rate of COPD-related HSU, including unscheduled physician and ED visits, and hospitalization. Spirometry was performed at the primary care site by the CRE according to the American Thoracic and European Respiratory Society (ATS/ERS) quality standards.^42^ We have previously published our primary care spirometry quality results.^43^ The KoKo® portable spirometer nSpire Health™ (Longmont, CO, USA) was used, and spirometry predicted values were from the National Health and Nutrition Examination Survey reference standards.^44^ Unscheduled urgent physician visits for COPD were confirmed by EMR chart audit. ED visits and hospitalization data were obtained from the Southwest Physicians’ Office Interface to Regional EMR (SPIRE) system, a provincial eHealth automated notification system.^45^

### Patient and public involvement

Patients were not directly involved in the development of the research question, study design, selection of outcome measures, conduct of the study, or recruitment. However, the patient perspective was actively solicited from our CRE network, and considered in all aspects of the study design and planning. This input influenced the selection of a patient-reported outcome (QoL) as our primary outcome. Patient participants will receive a copy of the published manuscript.

### Statistical analysis

Data were analyzed using a complete case analysis approach with SAS 9.4 (SAS Institute Inc., Cary, NC, USA). Data were expressed as mean (SD) for continuous values. For continuous variables, including the difference in the change in CAT score, CCQ score, Bristol Knowledge Questionnaire score, FEV1, and FEV1/FVC ratio, between-group differences were obtained via analysis of covariance adjusting for the baseline and the participating health centre. For dichotomous endpoints, including the proportion of individuals with a change in CAT score greater than the MCID, HSU, and COPD exacerbations reported as the proportion of subjects with “one or more” events, comparisons were made using Mantel–Haenszel chi-square tests and the associated common risk differences. A constrained longitudinal data analysis was conducted on the CAT score as a sensitivity analysis to evaluate the impact of missing data from patients lost to follow up.

Assuming a SD of 5.6, a sample size of 92 subjects per group was required to detect a difference of 3.0 in CAT scores with a two-sided 0.05 significance level with 90% power and allowing for a 20% loss to follow-up.

### Reporting summary

Further information on research design is available in the Nature Research Reporting Summary linked to this article.

## Supplementary information

Supplement 1: Asthma and COPD Point of Service System (POSS)

Life Sci Reporting Summary

## Data Availability

Individual de-identified participant data including the data dictionary will be shared including demographics, and primary and secondary outcomes data. The data will become available after peer reviewed publication of this manuscript and will remain available for 10 years. Data will be transmitted securely in SAS or Excel format following the execution of a data sharing agreement acceptable to the research ethics board. Data will be shared with university-based academics for use in systematic reviews. Data requests should be directed to clicskai@uwo.ca.

## Supplementary information

**Supplementary information** accompanies the paper on the *npj Primary Care Respiratory Medicine* website (10.1038/s41533-019-0119-9).
