# Supplementary material for: The impact of integrated disease management in high-risk COPD patients in primary care
Source: NPJ Prim Care Respir Med. 2019 Mar 28;29:8. doi: 10.1038/s41533-019-0119-9 (PMC6438975; doi:10.1038/s41533-019-0119-9)

**Supplement 1: Asthma and COPD Point of Service System (POSS)**

The first use of the COPD POSS was in the COPD Integrated Disease Management randomized controlled trial. The description below outlines the POSS functionality and current utilization.

**Brief Description of the POSS**

To facilitate quality improvement, quality assurance, scalability, and program evaluation, Asthma Research Group (ARGI) and the University of Windsor have collaboratively developed and implemented an electronic point-of-service system (POSS) for asthma and COPD that is currently utilized by all certified respiratory educators (CREs) at all participating primary care lung health program sites as an integrated information technology solution. Currently, the ARGI asthma and COPD programs are implemented in multiple sites across the Erie St. Clair Local Health Integration Network including 7 Family Health Teams (FHTs) (17 sites), 25 independent physicians, a Nurse Practitioner lead clinic, and 1 hospital based clinic. ARGI supports 670 full-day clinics /year, 3,000 patient visits, provided by more than 100 physicians/nurse practitioners and 11 CREs.

The POSS prompts the delivery of evidence-based best practices, records their delivery, and standardizes interventions across sites. It is adaptable in a cost effective manner based on new best practices, medications, or performance measures. There is extensive data checking at the time of data entry. Data definitions are incorporated to support quality data inputs. The POSS has the capability to easily extract hundreds of data-defined variables including key health system and patient performance metrics and to produce and deliver reports for providers and health administrators. All Ministry of Health and Long-Term Care program quarterly reports for the regional program are automated. Performance metrics are continuously updated and can be automatically extracted, and securely transmitted upon request or on a scheduled basis.

**POSS System Security:**

The POSS is built on a Windows 2008 R2 Server (Enterprise) environment, secured with up-to-date anti-malware and anti-viral protection, with scheduled updates on the threat definitions. The ARGI server resides in the secure physical server floor of the University of Windsor, with monitored key-card physical access controls, video-surveillance monitoring and alarms, fire-protection, UPS support, environmental controls, and logical security that includes network intrusion detection and advanced firewall protection with scheduled updating and maintenance windows on a regular basis. All data exchanges require encryption and secure network access controls. IBM Domino Server provides a robust PKI client to server relationship and thick client access requires certificate based access controls, secure socket layer networking and strong user application passwords. Server back-ups are automated incremental nightly back-ups, complemented with full weekend back-ups, and all back-ups are to a remote secure location using secure network encrypted exchange and storage. The applications developed use IBM's Notes Domino enterprise application architecture, with an integrated LDAP for authentication by user name and encrypted passwords. All passwords and data on servers are encrypted and not readable to system administrators. The network that provides services to the applications, as well as the server room have had both a Privacy Impact Assessment (PIA) and a Threat Risk Analysis (TRA) completed to ensure that the switching and router hardware, and outside plant meet the requirements of the Ontario Information and Privacy Office, I&IT Strategy, Policy, Planning and Management Branch, Office of the Corporate Chief Strategist, Management Board Secretariat. Government vendors of record were the only entities permitted to complete the TRA and PIA audits.

**The Provider Experience**

The POSS is used by CREs during every patient encounter as an integrated IT tool. It is perceived positively by the CRE as a patient support and quality improvement tool. The POSS is presented in a tabbed format including the following tabs: Authorization (permissions and consents), Demographics, Medical History, Health Contacts (health service utilization), Smoking History, Allergies, Medications, Assessment, and Evaluation. (Supplemental Figure 1) Within each tab there multiple data elements with mandatory fields, drop down menus, and radio buttons, minimizing free text entries. There are approximately 300 total data elements.

The Medications tab is below as Supplemental Figure 2. It highlights the drop down menu and radio button design. Hovering over a highlighted blue text area will activate the presentation of a data definition. A screen shot of the Skills Training and Educational Objectives Summary page is presented below as Supplemental Figure 3.

The POSS completes specific calculations based on data entered including: BMI, current age, GOLD classification and severity (based on MRC or CAT score and exacerbation / hospitalization profile), smoking pack-year history, and spirometry predicted values. Modest clinical decision support is provided in real-time. For example, after calculating the GOLD classification, the POSS will provide the GOLD based treatment recommendations back to the provider. (Supplemental Figure 4) Similarly, if the patient has a low BMI the POSS will provide a prompt for the CRE to consider referral to a dietician.

Supplement Figure 1: Tabbed Format with Diagnostics Section


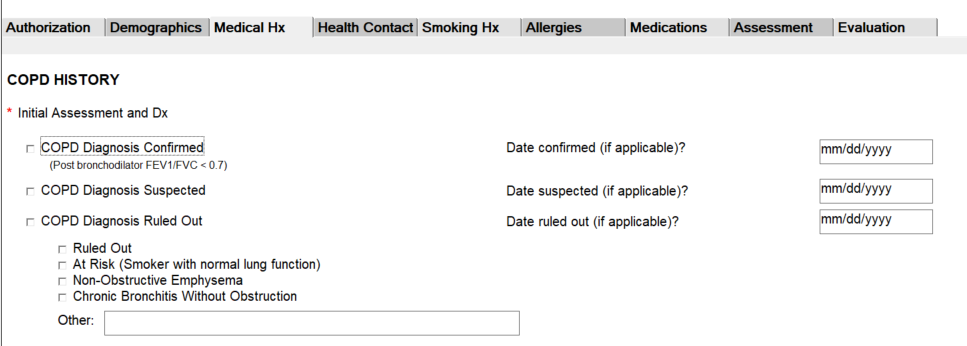


Supplement Figure 2: Medications Section


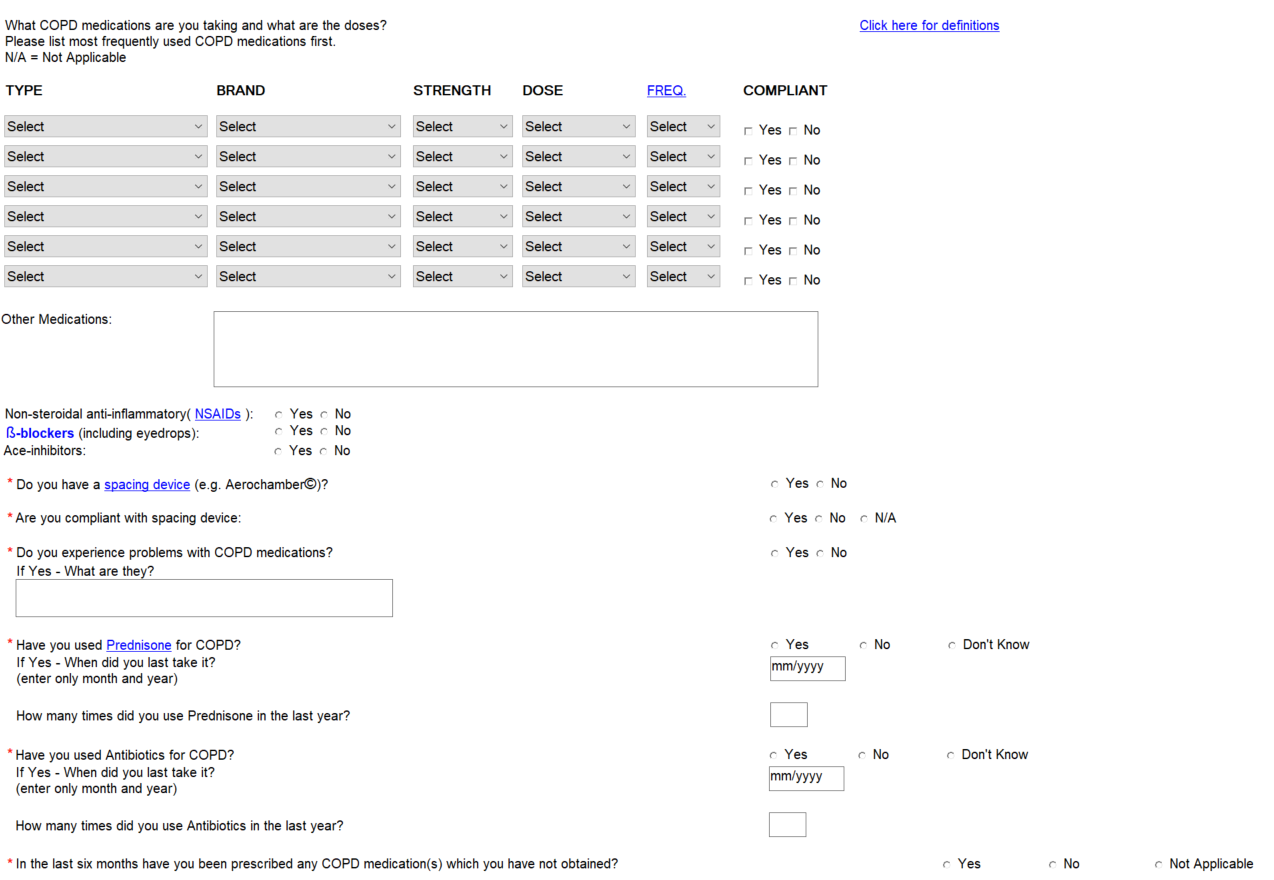


Supplement Figure 3: Skills Training and Educational Objectives Summary


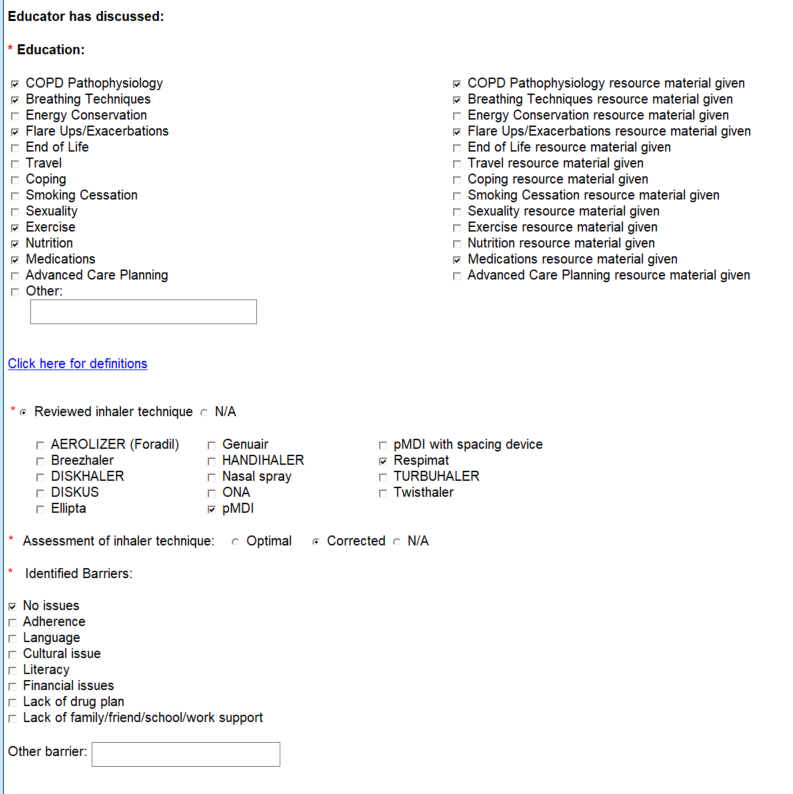


Supplement Figure 4: GOLD Risk Calculation and GOLD Treatment Recommendations


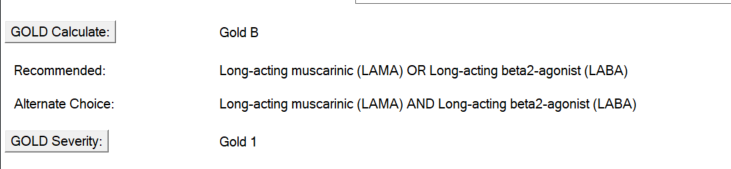

Supplement: Supplementary file 1 — Supplement 1: Asthma and COPD Point of Service System (POSS) [file 41533_2019_119_MOESM1_ESM.docx]
